# Non-Mouse-Adapted H1N1pdm09 Virus as a Model for Influenza Research

**DOI:** 10.3390/v12060590

**Published:** 2020-05-29

**Authors:** Irina Kiseleva, Andrey Rekstin, Mohammad Al Farroukh, Ekaterina Bazhenova, Anastasia Katelnikova, Ludmila Puchkova, Larisa Rudenko

**Affiliations:** 1 Department of Virology, Federal State Budgetary Scientific Institution “Institute of Experimental Medicine”, 197376 St Petersburg, Russia; arekstin@yandex.ru (A.R.); mouhammad1farroukh@gmail.com (M.A.F.); sonya.01.08@mail.ru (E.B.); puchkovalv@yandex.ru (L.P.); vaccine@mail.ru (L.R.); 2 Department of Toxicology and Microbiology, Institute of Preclinical Research Ltd., 188663 St Petersburg, Russia; katelnikova.ae@doclinika.ru

**Keywords:** influenza virus, influenza infection, animal model, viral toxicity, viral pathogenesis

## Abstract

The number of lung-adapted influenza viruses is limited. Most of them are not antigenically related to current circulating viruses. Viruses similar to recent strains are required for screening modern antiviral compounds and studying new vaccine candidates against novel influenza viruses. The process by which an influenza virus adapts to a new host is rather difficult. The aim of this study was to select a non-adapted current virus whose major biological properties correspond to those of classical lab-adapted viruses. Mice were inoculated intranasally with non-lung-adapted influenza viruses of subtype H1N1pdm09. They were monitored closely for body weight loss, mortality outcomes and gross pathology for 14 days following inoculation, as well as viral replication in lung tissue. Lung-adapted PR8 virus was used as a control. The tested viruses multiplied equally well in the lower respiratory tract of mice without prior adaptation but dramatically differed in lethality; the differences in their toxicity and pathogenicity in mice were established. A/South Africa/3626/2013 (H1N1)pdm09 virus was found to be an appropriate candidate to replace PR8 as a model virus for influenza research. No prior adaptation to the animal model is needed to reach the pathogenicity level of the classical mouse-adapted PR8 virus.

## 1. Introduction

Influenza A mouse and ferret models are widely used in influenza virus research in, for instance, preclinical studies of vaccine candidates to evaluate their safety, immunogenicity and efficacy in preventing influenza infection; to investigate the pathogenesis of influenza infection; or to screen antiviral compounds [1,2,3,4,5,6,7,8,9,10]. However, the susceptibility of the animal model to the influenza virus depends not only on the animal species but also on the particular virus strain to be tested in this model. The animal model needs to be exposed to the appropriate virus to adequately assess the safety, toxicity, pharmacodynamics and efficacy of any potential novel drug or vaccine.

Historically, the pathogenesis of influenza infection has been studied in mice using lab lung-adapted strains [2]. The limited number of classical mouse-adapted viruses includes A/Puerto Rico/8/34 (H1N1), B/Lee/40, A/Victoria/35/72 (H3N2), and A/Aichi/2/68 (H3N2). All of them are antigenically distinct from currently circulating viruses. However, a number of tests (for instance, the screening of antiviral agents) require the use of currently circulating strains.

The process by which an influenza virus adapts to a new host is rather difficult. Fortunately, certain influenza viruses of the avian origin [11,12], some H1N1 strains of the 2009 pandemic [13,14], and reconstructed 1918 Spanish influenza pandemic virus [15] may cause disease in mice without prior adaptation. For preclinical assessments of antiviral compounds or vaccine candidates, the currently circulating non-adapted virus could be a good choice.

It is important to understand that such a commonly used lab animal species like mice and ferrets may be fully sensitive to infection with non-adapted influenza viruses.

Primary viral pneumonia is recognized as the most severe pulmonary manifestation of influenza infection in mice inoculated with a mouse-adapted virus. Viral pneumonia develops on days 6–8 after intranasal infection. Conversely, non-mouse-adapted influenza viruses were shown to kill mice by exerting a so-called toxic effect when massive doses were administered [16]. Acute pulmonary edema develops on days 2–4 after the intranasal inoculation of mice. Both primary influenza pneumonia and toxicity have a high mortality rate, which can reach 100%. This study compared the in vivo properties of two non-mouse-adapted influenza viruses of the H1N1pdm09 subtype, namely, A/South Africa/3626/2013 and A/California/07/2009. The viruses were tested for their ability to cause sudden pulmonary edema and viral pneumonia. Two previous viruses that are antigenically non-related to currently circulating viruses, i.e., the mouse-adapted PR8 strain and non-mouse-adapted A/Singapore/1/57 (H2N2) strain, were used as controls.

## 2. Materials and Methods

### 2.1. Viruses

The following influenza A viruses were used: A/South Africa/3626/2013 (H1N1)pdm09 (SA), obtained from The Francis Crick Institute (London, UK); and the A/California/07/2009 (H1N1)pdm09 (CA), obtained from the CDC (Atlanta, GA, USA), CDC ID No. 2009712112; A/Singapore/1/57 (H2N2) (SGP) and four mouse-adapted strains—A/Puerto Rico/8/34 (H1N1) (PR8), B/Lee/40 (LEE), A/Victoria/35/72 (H3N2) (VIC), and A/Aichi/2/68 (H3N2) (ACH)—were obtained from the Virus Collection of the Institute of Experimental Medicine (IEM, St. Petersburg, Russia) (Table 1).

All the work with the influenza viruses was performed in a Class II biosafety cabinet (BSC) in a biosafety level-2 (BSL-2) laboratory.

The viruses were propagated in the chorioallantoic cavity of 10–11 day old clean chicken eggs supplied by «Sinyavino» poultry farm (Kirovsk Area, Leningrad region, Russia). The eggs were incubated for 48 h at 32 °C and the harvested viruses were stored in aliquots at −70 °C.

### 2.2. Determining ts/ca Phenotype

The capacity of influenza viruses to grow at optimal (32 °C), low (26 °C, cold-adapted virus, *ca* phenotype), and elevated (40 °C, temperature-sensitive virus, *ts* phenotype) temperatures was determined by titration in 10–11 day old eggs. The log_10_ 50% embryo infectious dose (EID_50_/mL) calculation was based on Reed and Muench’s method [17]. The viruses were considered as possessing a *non-ts* phenotype if log_10_ EID_50_/mL at 32 °C–log_10_ EID_50_/mL at 40 °C ≤ 4.0 log_10_ EID_50_/mL. The viruses were considered as having a *non-ca* phenotype if log_10_ EID_50_/mL at 32 °C–log_10_ EID_50_/mL at 26 °C ≥ 3.0 log_10_ EID_50_/mL.

### 2.3. Mice 

Female BALB/c mice of 6–8 weeks of age were supplied by the laboratory breeding nursery “Rappolovo” (St. Petersburg Region, Russia). The animals were allowed free access to food and water.

### 2.4. Ferrets

Male ferrets (*Mustela putorius furo*), aged 5–6 months and weighing 1.1–1.9 kg at the beginning of the experiment, were supplied by the Scientific Production Organization House of Pharmacy JSC (St. Petersburg, Russia). They were prescreened by a routine hemagglutination inhibition test to ensure that they were negative for the A/South Africa/3626/2013 (H1N1)pdm09 virus being tested. Prior to infection, the ferrets were randomly divided into two groups and housed individually in isolation units with free access to food and water. The unpublished results of the testing ferrets nested in a past preclinical trial [18] are included in this paper. The ferrets were monitored for clinical signs and morbidity. The body temperature of the animals was recorded by temperature loggers (Data Storage Tag, DST micro-T ultra-small temperature loggers; Star-Oddi, Reykjavik, Iceland), which were placed in the peritoneal cavity.

### 2.5. Ethics Statement

The mice, ferrets and chicken embryos were handled in accordance with European Union legislation [19] and the Russian Manual for laboratory animals [20]. The mice use protocol (No. 1/20) was approved on 27 February 2020 by the Institutional Local Ethical Committee (IEM, St. Petersburg, Russia). The ferret study was conducted using protocol No. BEC 2.12/18 approved on 28 February 2018 by the Local Bioethical Committee of the Institute of Preclinical Research Ltd. (St. Petersburg, Russia). At the end of the study, the animals were humanely euthanized. The fertilized eggs used for virus propagation were discarded in an appropriate manner, according to the Russian Sanitary epidemiological rules SP 1.3.2322-08 (approved 28 Jan 2008).

The 50% lethal dose (LD_50_), 50% mouse infectious dose (MID_50_), and 50% pneumonia dose (PnD_50_) in mice were evaluated to standardize the infectious dose of the influenza viruses that possess different properties. The lethal dose was determined as the dose of the virus that kills 50% of the animals, MID_50_ was determined by isolating the virus from animal lungs, and PnD_50_ was determined by assessing the severity of gross pathological lesions in lungs on day 14 after infection.

The 50% dose was calculated by the routine Reed and Muench method [17] and expressed as log_10_ EID_50_/mL. Under inhalation anesthesia by ether, the animals were infected intranasally with serial 10-fold dilutions of the tested virus in 50 μL of phosphate-buffered saline (PBS) containing the appropriate viral dilution, divided equally between the nostrils.

### 2.6. Viral Replication in Lung Tissue

To determine the virus infectivity in the lower respiratory tract, the mice were lightly anesthetized with ether and inoculated intranasally with 50 μL of PBS containing 10^2^ MID_50_ of the influenza virus, divided equally between the nostrils. The viral load was measured in lung samples collected on day 3 post infection. Tissue homogenates were prepared using a small bead mill TissueLyser LT (QIAGEN, Germany) in 1.0 mL of PBS containing antibiotic-antimycotic (Invitrogen, UK); the clarified supernatants were titrated in eggs at the temperature of 32 °C.

Ferrets (five animals per group) were given 6.0 log_10_ EID_50_/mL of SA or PBS. The preparations were divided between the two nostrils and given to the animals under short-term anesthesia induced by the intramuscular injection of Zoletil 100 at a dose of 12.5 mg/kg of body weight. The samples of the lung tissue of ferrets were taken on day 4 after infection. Nasal washes were collected until 4 days after infection. All the samples were analyzed for the presence of virus by culturing in embryonated chicken eggs, as described above.

### 2.7. Gross Pathology

A complete macroscopic post-mortem (gross pathology) examination was performed at the time of death or at the end of the observation period of 14 days. All the lung lobes were inspected. Macroscopic changes in the lungs were assessed in accordance with the color and the severity of the lesions.

### 2.8. Acute Toxicity in Mice (Development of Acute Pulmonary Edema)

A massive dose of virus (≥8.0 log_10_ EID_50_) was administered intranasally in mice under inhalation anesthesia by ether. The lethality from acute pulmonary edema was monitored daily for the first 4 days post-infection.

### 2.9. Phylogenetic Analysis

Phylogenetic analysis of H1N1pdm09 viruses was performed using the nextstrain.org website [21].

### 2.10. Statistics

Statistical analysis of the results was carried out using the GraphPad Prizm 7 and Statistica 10 software. The non-parametric Mann–Whitney test and Kruskal–Wallis test were applied for data comparison. A *p*-value < 0.05 was considered to be statistically significant.

## 3. Results

### 3.1. Determining ts and ca Phenotype In Ovo

The capacity to replicate outside of the optimal temperature of incubation is one of the important phenotypic characteristics of wild-type (WT) viruses. Four WT viruses (two antigenic variants of H1N1pdm09, reference virus of the Asian pandemic of 1957, and PR8) were studied for their capacity to grow at temperatures of 32 °C (optimal temperature), 26 °C and 40 °C.

*Ts* and *ca* characteristics of the H1N1pdm09 viruses were similar to those of previous pandemic and epidemic strains, SGP and PR8, respectively. Regardless of their antigenic characteristics, the reference virus of the Asian pandemic (SGP), two novel H1N1pdm09 viruses (CA and SA), and PR8 displayed the same pronounced *non-ts/non-ca* phenotype as the corresponding typical WT influenza virus (Table 2). The viruses were able to grow at an elevated temperature of 40 °C and were not able to grow at 26 °C.

### 3.2. Viral Replication in Lung Tissue

The replication capacity of different influenza viruses in the lungs of mice was evaluated. The influenza virus replication in the lower respiratory tract was detected, despite differences in antigenic specificity and their level of adaptation to mice. On day 3 post administration of 10^2^ MID_50_, the lung virus titers of PR8 and SA reached 8.8 ± 0.5 log_10_ EID_50_/mL/g tissue and 8.9 ± 0.3 log_10_ EID_50_/mL/g tissue, respectively. The lowest lung virus titer was detected for CA (4.2 ± 0.4 log_10_ EID_50_/mL/g tissue) (Table 2). Although CA replicated in the lower respiratory tract of mice, it did not cause severe lung lesions that are typical of infection with the mouse-adapted PR8 strain.

The low dose of PR8 (1.0 log_10_ EID_50_/mL) infected 50% of the tested mice. Similarly, the low dose of SA (1.8 log_10_ EID_50_/mL) caused detectable virus replication in the lung tissue of 50% of the infected mice (Table 2). In contrast, to infect 50% of the mice with SGP and CA, higher doses were needed; 1.0 MID_50_ was found to be 5.5 log_10_ EID_50_/mL and 6.0 log_10_ EID_50_/mL, respectively.

### 3.3. Acute Toxicity in Mice

**Figure 1:**
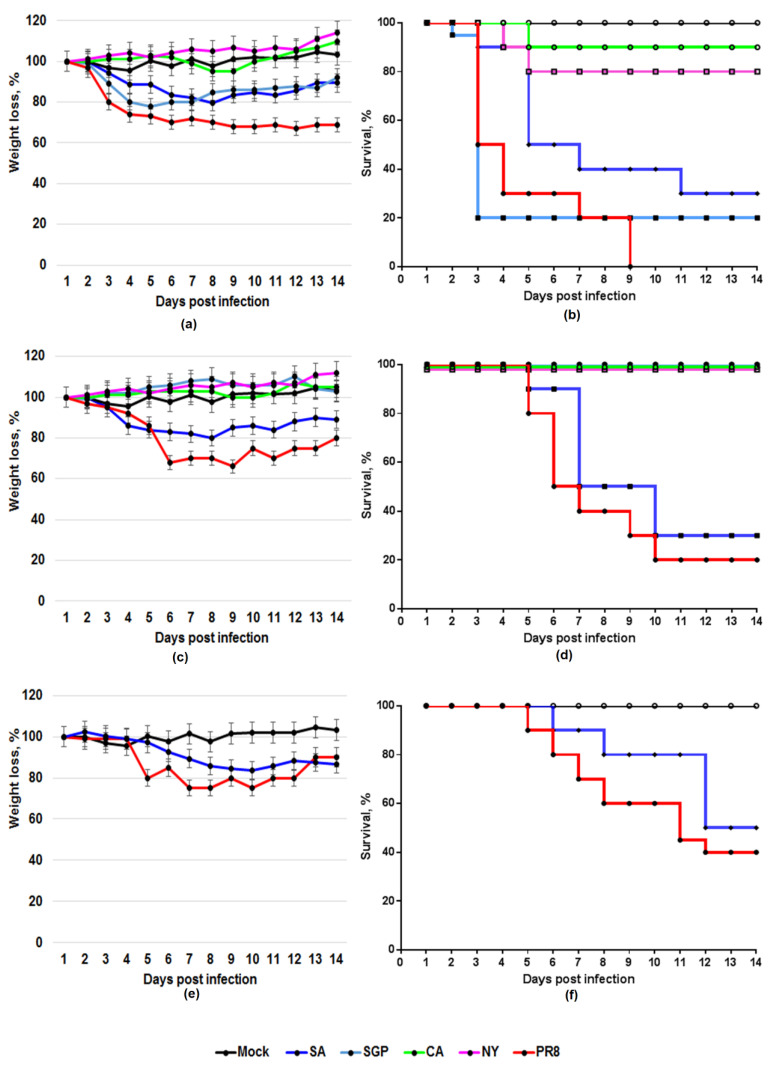
Weight loss and lethality of the influenza viruses for BALB/c mice. Weight loss, %, *p* < 0.05 (**a**,**c**,**e**), and survival, % (**b**,**d**,**f**), were monitored daily for 14 days. Mice were inoculated intranasally with 8.5 log_10_ EID_50_ (**a**,**b**), 7.5 log_10_ EID_50_ (**c**,**d**) or 5.0 log_10_ EID_50_ (1–10 LD_50_) (**e**,**f**).

Massive doses of SGP, PR8 or SA were administered intranasally to mice causing 70%–80% mortality during the first days post infection (Table 2, Figure 1b).

**Figure 2:**
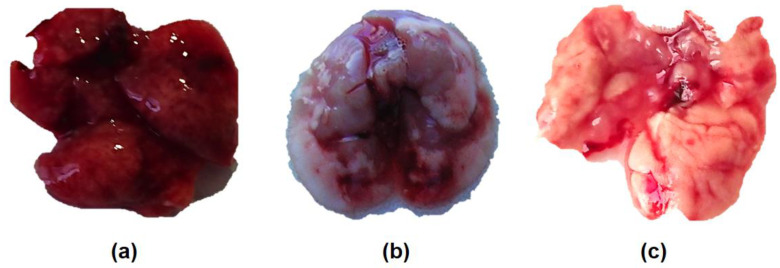
Gross examination of the lungs of mice infected with A/South Africa/3626/2013 (H1N1)pdm09 influenza virus: (**a**) hemorrhagic pulmonary edema (8.2 log_10_ EID_50_/mouse, D4); (**b**) primary viral pneumonia (1 PnD_50_/mouse = 5.0 log_10_ EID_50_/mouse, D14); (**c**) normal lungs of a control mouse inoculated with phosphate-buffered saline (PBS) (D14).

The pathological examination of the lungs of dead mice showed that acute pulmonary edema affected all lobes of the lung. At the time of toxic death, the lungs were hemorrhagic (Figure 2a).

### 3.4. Pathogenicity in Mice

**Figure 3:**
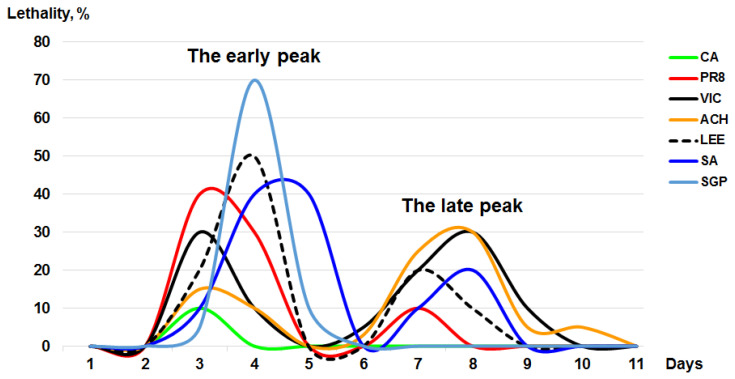
Lethality of the influenza viruses for the BALB/c mice. The mice were inoculated with a massive dose of influenza virus (8.0–9.0 log_10_ EID_50_/mL).

Mice were intranasally inoculated with a 10-fold serial dilution of each virus in PBS under general anesthesia with ether. The weight changes and survival rates were monitored daily for two weeks. The results are summarized in Table 2 and Figure 3. The viruses differed in the main characteristics of pathogenicity being tested in the mice. As shown in the section “acute toxicity in mice”, the toxic action of the intranasally inoculated mouse-adapted PR8 influenza virus killed mice within the first 3–4 days. As can be seen from Figure 3, the second peak of late deaths occurred at 6–10 days post infection and was due to pulmonary consolidation and primary viral pneumonia. Interestingly, non-mouse-adapted SA was also shown to be pathogenic to mice.

As reported in Figure 3, we found one peak of toxic death at 2–4 days post infection in mice that were administered a high dose of SA and the second one at 6–10 days. For SA, 50% LD was found to be 4.2 log_10_ EID_50_/mL. Lung-adapted PR8 displayed comparable pathogenicity: LD_50_ was found to be 4.0 log_10_ EID_50_/mL. Another non-mouse-adapted strain, SGP, caused acute pulmonary edema when administered intranasally at the high dose. It killed 90% of mice in 4 days. However, unlike SA, it has no natural ability to adapt to mice.

The ability of the non-adapted SGP to cause severe lung lesions disappeared with the virus dilution. The results indicated that one log_10_ reduction in the infectious dose resulted in the loss of the ability to cause toxic pulmonary edema. When diluted 10 times, the SGP did not display pathogenicity for mice; all the animals survived (Figure 1d). In contrast, when inoculated into mice at the low dose, SA maintained its pathogenicity. When administered as a dose of 1–10 LD_50_ (5.0 log_10_ EID_50_/mL), SA caused a lethal infection in mice compared with that of the PR8.

The maximum weight loss analysis revealed that the difference in body weight loss between the tested viruses was determined primarily by differences in their ability to cause lethal infection in mice. Weight loss (Figure 1e) and the level of lethality (Figure 1f) of mice infected with 1–10 LD_50_ of PR8 were compared with those of SA. Both the mouse-adapted PR8 and non-adapted SA strains caused severe weight loss compared with a mock group when a dose of 1–10 LD_50_ (5.0 log_10_ EID_50_/mL) was administered intranasally to the BALB/c mice. CA, which is not lethal to mice, did not cause significant weight loss.

### 3.5. A/South Africa/3626/2013 Virus Infection in Ferrets

**Figure 4:**
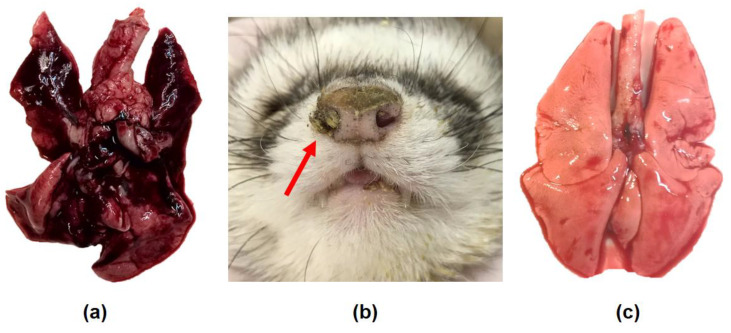
Nasal symptoms and gross examination of the lungs of the ferrets infected with A/South Africa/3626/2013 (H1N1)pdm09 influenza virus. (**a**,**b**) The ferrets were inoculated with 6.0 log_10_ EID_50_. (**a**) Severe lung lesions were registered on day 4 post infection. (**b**) On D3 post infection, the animals displayed sneezing, nasal discharge on the external nares (red arrow) and mouth breathing. (**c**) In contrast, the lungs of the ferrets inoculated with PBS were normal.

The non-lung-adapted SA influenza virus in a dose of 6.0 log_10_ EID_50_/mL caused sneezing, nasal discharge on the external nares, and mouth breathing on day 3 post administration (Figure 4b) and severe lung lesions on day 4 (Figure 4a).

**Figure 5:**
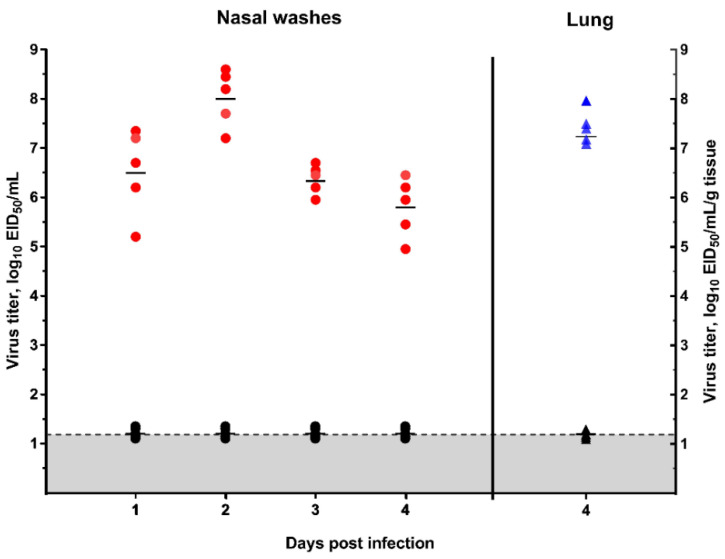
Virus replication in the lungs and upper respiratory tract of the ferrets as measured by titration in embryonated chicken eggs (mean ± SD).

The virus efficiently multiplied in the lower respiratory tract of the ferrets. This was estimated through the titration of lung tissue samples taken on day 4 post infection (Figure 5). A virus titer of 7.3 ± 0.4 log_10_ EID_50_/mL/g lung tissue was achieved. On day 2 post infection, the virus titer reached a maximum titer of 8.0 ± 0.4 log_10_ EID_50_/mL.

Red circle—virus titer in the nasal washes of the infected ferrets; blue triangle—virus titer in the lung tissue of the infected ferrets; black circle—virus titer in the nasal washes of the ferrets inoculated with PBS; black triangle—virus titer in the lung tissue of ferrets inoculated with PBS; gray—the limit of virus detection (1.2 log_10_ EID_50_/mL).

**Figure 6:**
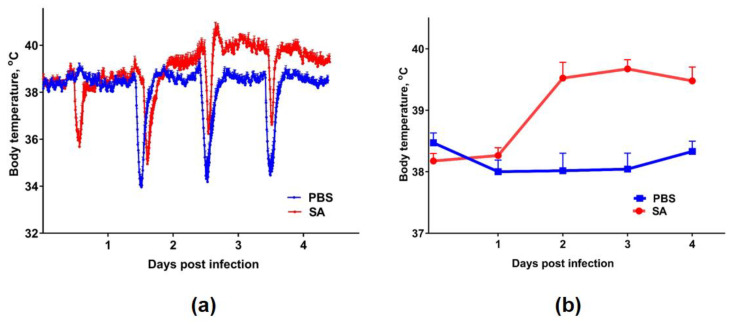
Body temperature of the ferrets after infection with the SA influenza virus: (**a**) variation in the body temperature until 4 days post infection (raw data). The temporary decreases in temperature were the result of sedation during the collection of nasal washes. The body temperature was recorded by temperature loggers every 10 min; (**b**) the difference between the infected group and the control group, represented by the mean ± SD, *p* < 0.05.

The body temperature of the ferrets was recorded on days 0–4 every 10 min (Figure 6a). A significant rise in body temperature (1.5–2 °C, average 40 °C) was seen in the animals after the inoculation with SA influenza virus compared with the control group, which was administered PBS (Figure 6b). These data confirm the development of the disease in the inoculated animals.

### 3.6. Phylogenetic Characterization of H1N1pdm09 Viruses

**Figure 7:**
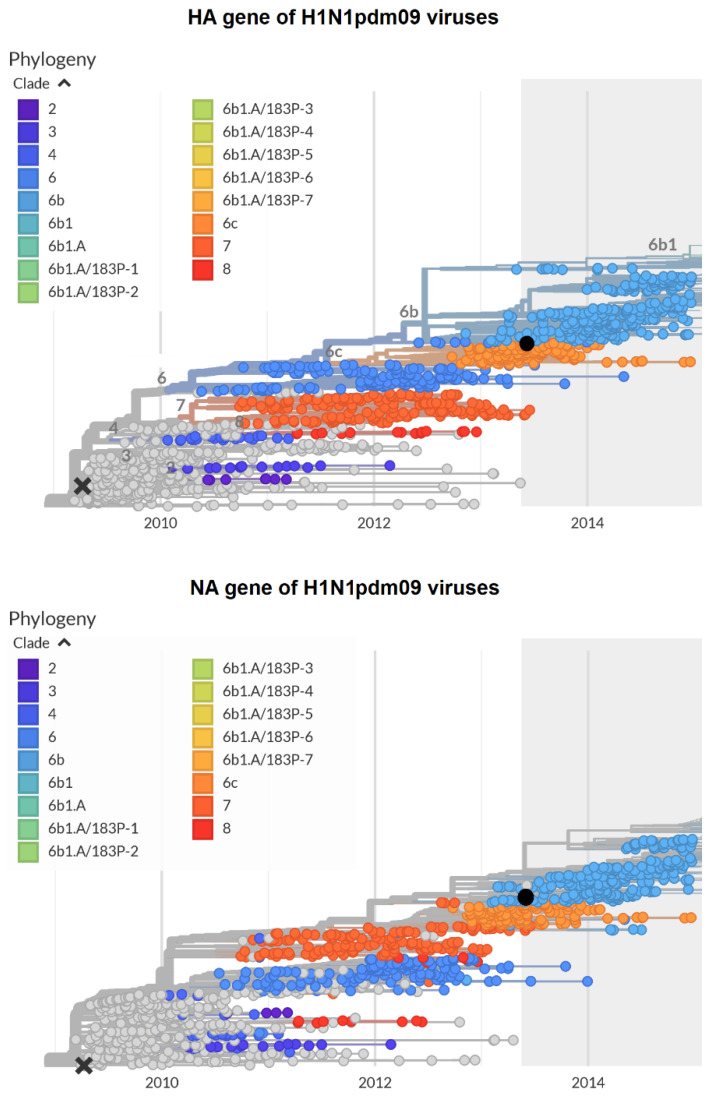
Phylogenetic trees of hemagglutinin (**upper panel**) and neuraminidase (**lower panel**) of the H1N1pdm09 influenza viruses. The website https://nextstrain.org/ [21] was used to construct the figure. Black cross: A/California/07/2009; black circle: A/South Africa/3626/2013.

The analysis of the H1N1pdm09 viruses that circulated between 2009 and 2014 revealed that the HA and NA of the A/California/07/2009 reference virus were genetically distinguished from those of A/South Africa/3626/2013 (Figure 7). For instance, the following mutations were identified in the HA2 of A/South Africa/3626/2013 compared with A/California/07/2009: S83P/I321V, S203T E47K, D97N, S185T/S124N, K283E/E172K, K163Q/A256T [22].

## 4. Discussion

The number of mouse-adapted lab influenza viruses is limited. The first mouse-adapted strains were initially developed in the 1930s–1960s. The most famous of them, PR8, was isolated in 1934. By 1938, the PR8 strain had already been passaged in mice 158 times [23]. In 1940, the number of mouse lung-to-lung passages had already reached 333 [24]. In addition, it has had over 100 passages in ferrets [25] and 200 passages in embryonated chicken eggs [26]. According to another source, the adaptation process of PR8 involved 77 passages in mice; 717 passages in cell culture; 30 passages in chicken embryos; five passages in ferrets; and an additional 50 passages in chicken embryos [27]. The result of such a long passage history was the complete attenuation of the virus for humans and a high level of adaptation to mice. PR8 has been used for pathogenicity studies for decades; however, this virus and the currently circulating H1N1pdm09 strains are antigenically and genetically dramatically distinct H1N1 viruses. Other classical mouse-adapted viruses are also antigenically out-of-date.

In recent years, numerous antigenically relevant human-origin or avian-origin influenza viruses have been adapted to other hosts. Some of them were adapted by sequential lung-to-lung passages through the new host [28,29,30,31,32], and others were obtained by introducing host-range mutations [32,33,34]. The adaptation of the influenza virus in a new host is rather difficult and may take time. However, it was recently shown that certain influenza viruses of human or avian origin might cause disease in mice without prior adaptation [11,12,13,14,15].

Therefore, in our study, an attempt was made to select a non-adapted current virus whose major biological properties corresponded to those of the lab-adapted virus. We compared the main biological properties of the classical mouse-adapted PR8 virus, reference virus of the Asian pandemic of 1957 SGP, and two current human influenza viruses of subtype H1N1pdm09. The objective of our study was to evaluate the possibility of using the current influenza virus for influenza studies without adaptation to a new host.

It has been previously shown that the influenza viruses that caused past pandemics or large outbreaks were typically able to grow at a temperature above the normal physiological range [35,36]. The *ts* and *ca* characteristics of the viruses tested in this study were similar to those of typical pandemic and epidemic WT viruses. All of them displayed a pronounced *non-ts/non-ca* phenotype: they were able to grow at an elevated temperature of 40 °C and not able to grow at 26 °C.

The standardization of the infectious dose is important for the study design and the subsequent interpretation of the results. The literature describes three options for standardizing the dose of the influenza virus to be used to infect experimental animals. (i) The first measure is the mouse infectious dose (MID_50_) [12,37]. Depending on the objectives, the animals were infected with 10^1^–10^2^ MID_50_. However, a preliminary determination of the MID_50_ is often an expensive procedure, especially when it comes to animals such as monkeys or ferrets. As an option, the infectious dose to be inoculated into animals can be established by (ii) the measure of the infectious virus titer in eggs (fifty-percent embryo infectious dose, EID_50_) or in tissue culture (fifty-percent tissue culture infectious dose, TCID_50_; plaque-forming units, PFU); the animals are infected with a dose in the range of 5.0–7.0 log_10_ EID_50_ [38,39,40,41,42,43] or 3.0–8.0 log_10_ TCID_50_/PFU [7,44,45,46,47,48,49]. (iii). If the virus to be tested is lung-adapted and may cause animal death, the fifty-percent lethal dose (LD_50_) can be used [2,37,46].

In this work, the mice experiments were conducted by determining the EID_50_, MID_50_, LD_50_ and the rarely defined PnD_50_. For the ferret study, to reduce pain and the number of experimental animals, we followed the principles of the 3Rs (replacement, reduction and refinement) and reduced the number of experimental groups to two: group 1 was inoculated intranasally with one dose of the WT virus—6.0 log_10_ EID_50_; group 2 was inoculated with the placebo (PBS).

In mammalian models (mice), the 2009 pandemic H1N1 influenza viruses that have evolved since the pandemic onset were not able to induce interstitial pneumonia [28]. In our experiments, the first 2009 pandemic virus, A/California/07/2009, efficiently replicated in the lower respiratory tract of mice but did not cause the severe lung lesions that are typical of infection with a mouse-adapted PR8 strain. In contrast, during their circulation in humans, some H1N1pdm09 viruses have acquired the pronounced ability to cause fatal pneumonia in mice.

The phylogenetic analysis of the HA genes of A(H1N1)pdm09 viruses circulating between 2009 and 2014 revealed a number of genetic groups, in which the reference strain A/California/07/2009 represents group 1 (clade 1). Between the pandemic 2009 A/California/07/2009 and A/South Africa/3626/2013 viruses (clade 6B), multiple substitutions have emerged in HA [22], resulting in viruses that possess new biological properties, such as a high toxicity and pathogenicity in lab animals. SA, isolated in 2013, was shown to be very toxic and pathogenic to mice. The 50% lethal dose of non-mouse-adapted SA was found to be 4.2 log_10_ EID_50_/mL. For comparison, the 50% lethal dose of mouse-adapted PR8 was found to be very close to that of SA (4.0 log_10_ EID_50_/mL).

Influenza viruses were shown to kill mice by exerting a so called toxic effect. The acute toxicity of mouse-adapted influenza viruses was discovered in the 1940s [16,50]. It has been reported that a massive dose of the virus, when inoculated by intranasal or intravenous routes, may cause severe lesions and damage in the lungs of mice. This toxic effect was not produced unless at least 8.0–8.5 log_10_ EID_50_ of the virus was inoculated [51]. The toxic property of influenza viruses was studied extensively in the 1940s–1960s. However, the nature and detailed mechanisms of this phenomenon remain unclear.

In the latter study, after intravenous injection, the PR8 virus killed rabbits [52]. Apparently, the mouse-adapted virus may be considered non-rabbit-adapted. In another study, the death of mice from sudden pulmonary edema occurred after they were infected with a very high dose of the non-mouse-adapted influenza virus [53].

In our experiments, the toxic effects of the intranasally inoculated lung-adapted PR8 influenza virus killed mice within the first 3–4 days. After these toxic deaths, the second peak of late deaths was detected at 6–10 days and was due to primary viral pneumonia. These results correspond to early publications [51]. Conversely, only one peak of early deaths (D2–D4) was seen after the inoculation of mice with non-mouse-adapted viruses. Ninety percent and 80% of the mouse deaths were recorded in the first 4 days post-intranasal inoculation with the non-mouse-adapted SGP and SA, respectively.

Two tested H1N1pdm09 viruses differed in their ability to cause acute pulmonary edema. CA did not cause pronounced toxic effects in BALB/c mice (10% lethality by day 4 post-intranasal administration). In contrast, the lethal effect of the intranasal inoculation of the massive dose of SA or SGP in mice was rather strengthened, with 20% and 10% survival by day 4 post infection, respectively. The lungs of the dead mice were examined for gross pathological lesions. This examination revealed pronounced acute pulmonary edema.

We found that the non-mouse-adapted SA virus displayed pathogenicity that was comparable to that of the mouse-adapted PR8 virus. This notion was supported by the observation that the main characteristics of SA (toxicity and lethality for mice, the level of virus replication in lungs, *non-ts/non-ca* phenotype) were close to those of PR8. Mice and ferrets are the most employed animals in virology. Therefore, it is important that SA is shown to be a good model for influenza research in both these animal models.

Intoxication is a typical clinical symptom of influenza. The significant association between severe manifestations of influenza infection and the toxicity of the influenza virus is worth further investigation. To facilitate the development of antivirals against acute pulmonary edema of influenza virus origin, screening platforms based on non-adapted, highly toxic influenza viruses should be established.

It is of importance to identify which mutations determine virulence, pathogenicity and the host range of mammals. The mechanisms responsible for a virus’ adaptation to a new host or its enhanced virulence are not fully understood. The contribution of genes coding HA, PB2, NP, or NA proteins to enhanced pathogenicity and mouse adaptation was established in a number of studies [32,54,55,56,57,58,59,60,61,62,63,64], which suggest that mouse-adapted changes in HA and PB2 are key factors in increased pathogenicity and the adaptation in a new host. The mechanism of the natural adaptation of SA to mice is not clear yet.

The HAs of the early 2009 H1N1 pandemic viruses are not able to maintain a trimeric complex when expressed in a recombinant system. In contrast, the hemagglutinins of recent strains are more stable; the improvement of their stability is attributed to an E47K substitution in the HA2 subunit of the stalk that emerged naturally in the currently circulating viruses [65,66]. The strains isolated after 2010 possess new HA substitutions near the HA receptor-binding site (N125D and D127E or D127E and K209E) and efficiently grow in embryonated chicken eggs and mammalian cell lines [67]. Hemagglutinin thermostability is an important property of the influenza virus. Highly pathogenic avian influenza viruses displayed a higher thermostability compared with viruses with low pathogenicity [68]. The resistance of HA to high temperatures contributes to environmental resistance in viruses [69] and affects pathogenicity in sensitive mammalian hosts [70]. Pathogenicity for animals of the SA may be associated with more pronounced thermostability of its HA.

## 5. Conclusions

We believe that the reported data set allows us to make the overall conclusion that currently circulating non-mouse-adapted influenza viruses may be used to evaluate potential antiviral and antitoxic drugs and vaccine candidates. The advantage of non-adapted viruses is that there is no need for additional lab work for their adaptation to model animals. Non-mouse-adapted A/South Africa/3626/2013 (H1N1)pdm09 virus is a good option to use as a model for influenza research.

## Figures and Tables

**Table 1:** Viruses used in this study.

Strain Designation	Type/Subtype	Lab Adaptation to Mice	Pathogenicity in Mice	Acute Toxicity in Mice
PR8	H1N1	yes	yes	yes
LEE	B	yes	yes	yes
VIC	H3N2	yes	yes	yes
ACH	H3N2	yes	yes	yes
SGP	H2N2	no	no	yes
CA	H1N1pdm09	no	no	no
SA	H1N1pdm09	no	yes	yes

**Table 2:** The main characteristics of the influenza viruses tested in vivo and in ovo.

Testing	Strain Designation			
	PR8	SGP	CA	SA
In Vivo Study				
Acute toxicity ^1^, %	8/10 (80%)	8/10 (80%)	1/10 (10%)	7/10 (70%)
LD_50_^2^	4.0	>8.5	>8.0	4.2
PnD_50_^3^	2.3	>8.5	>8.0	2.8
MID_50_^4^	1.0	5.5	7.0	1.8
Max weight loss ^5^, %	22.2	25.2	3.1	20.3
Mean virus lung titer ^5^ ± SD	8.8 ± 0.5	6.8 ± 0.4	4.2 ± 0.4	8.9 ± 0.3
In Ovo Study				
Mean virus titer ^6^ ± SD at 32 °C	9.0 ± 0.3	8.5 ± 0.2	8.0 ± 0.2	9.2 ± 0.5
Mean virus titer ^6^ ± SD at 40 °C	7.2 ± 0.3	6.5 ± 0.5	6.8 ± 0.3	8.7 ± 0.4
Mean virus titer ^6^ ± SD at 26 °C	2.2 ± 0.3	1.8 ± 0.1	2.0 ± 0.2	4.0 ± 0.3
Phenotype	*non-ts/non-ca*	*non-ts/non-ca*	*non-ts/non-ca*	*non-ts/non-ca*

^1^ Mortality from acute hemorrhagic pulmonary edema to day 4 post-intranasal inoculation of undiluted virus-containing allantoic fluid containing ≥ 8.0 log_10_ 50% embryo infectious dose (EID_50_/mL) of the virus. ^2^ Expressed as the log_10_ EID_50_/mL required to give 1 50% lethal dose (LD_50_). ^3^ Expressed as the log_10_ EID_50_/mL required to give 150% pneumonia dose (PnD_50_). ^4^ Expressed as the log_10_ EID_50_/mL required to give 1 50% mouse infectious dose (MID_50_). ^5^ Mice were inoculated with 100 MID_50_; expressed as log_10_ EID_50_/mL/gram of lung tissue. ^6^ Expressed as log_10_ EID_50_/mL.
